# Selective-Area Deposition of Indium and Its Plasmonic Properties

**DOI:** 10.1021/acsaom.5c00373

**Published:** 2025-12-10

**Authors:** Didem Dede, Evelijn Akerboom, Riccardo Brondolin, Tom Veeken, Thomas Hagger, Raphael Lemerle, Esther Alarcon Llado, Valerio Piazza, W. Craig Carter, Albert Polman, Anna Fontcuberta i Morral

**Affiliations:** † Laboratory of Semiconductor Materials, Institute of Materials, 27218 École Polytechnique Fédérale de Lausanne, Route Cantonale, Lausanne, Vaud 1015, Switzerland; ‡ Center for Nanophotonics, 55952 NWO-Institute AMOLF, 1098XG Amsterdam, The Netherlands; § Department of Materials Science and Engineering, 2167 Massachusetts Institute of Technology, Cambridge, Massachusetts 02139, United States; ∥ Institute of Physics, École Polytechnique Fédérale de Lausanne, Route Cantonale, Lausanne, Vaud 1015, Switzerland

**Keywords:** indium nanoparticles, selective area deposition, molecular beam epitaxy, plasmonics, cathodoluminescence

## Abstract

We present an effective process sequence for the deposition of indium nanostructures using molecular beam epitaxy (MBE) on a silicon substrate. Using a template structure composed of inverted pyramids and V-grooves, we deposit indium nanostructures with various dimensions. Spatially resolved cathodoluminescence spectroscopy (CL) using an electron-beam energy of 30 keV electrons shows a localized surface plasmon (LSP) resonance in spherical particles with a peak wavelength at 300 nm and a full width at half-maximum of 70 nm for the smallest particles (diameter of 85 nm), showing high optical quality of the grown indium. V-groove template structures create indium nanowires for which CL spectroscopy reveals efficient propagation of surface plasmon polaritons (SPPs), and angle-resolved CL on the periodic inverted pyramids reveals optical lattice resonances arising from the array’s periodicity. The high optical quality of these nanostructures enables further applications of plasmonic nanostructures in the ultraviolet (UV) spectral range.

## Graphical abstract

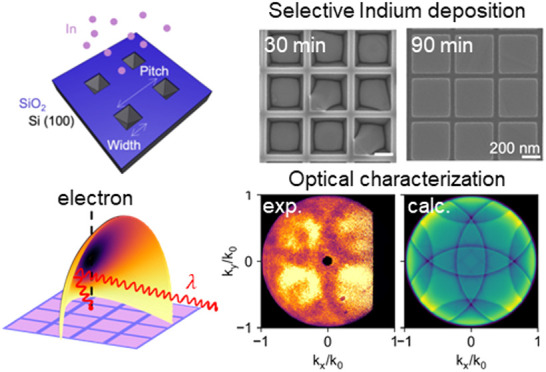

## Introduction

Noble metal nanostructures show strong interaction with light due to collective oscillations of the free electrons in their conduction band. When excited, they can support surface plasmon polaritons (SPPs) and localized surface plasmons (LSPs).^1–3^ Metal nanogrids, for instance, can function as transparent conductors for photovoltaics.^4^ Furthermore, SPPs can serve as information carriers in integrated optical elements with length scales well below the optical diffraction limit, while LSPs exhibit strong absorption and scattering resonances in the ultraviolet (UV)–visible-near-infrared spectral range. These resonances enable a variety of applications in, for example, sensing, photo, or thermally enhanced catalysis driven by strong optical near-fields.^5^ The LSP resonance wavelengths are tunable with particle size, morphology, metal composition, and the surrounding dielectric environment. This tunability offers significant design flexibility, providing a versatile platform for exploiting plasmonic properties and enriching applications involving metal nanostructures.^6^

Assembling multiple metal nanoparticles into ordered arrays further enables advanced functionalities. When metal particles are positioned at controlled distances, the coherent interaction between excited plasmons can create strong optical near-fields, and as a result, controlled coupling to the far-field.^1,7–9^ Various methods enable the formation of plasmonic lattices. One favorable bottom-up technique is a sequence of substrate patterning, metal deposition at exposed areas by evaporation or sputtering, and lifting off the residual resist.^10^ This relatively simple approach enables the formation of regular arrays of metal nanostructures with defined distances, sizes, thicknesses, and morphologies. Colloidal self-assembly is another method to form nanoparticle arrays.^11,12^

Noble metal nanostructures composed of silver, gold, and copper are among the most extensively studied and exhibit optical resonances in the visible and near-infrared spectral range.^13–15^ Aluminum, a non-noble metal, displays a resonance in the visible to deep ultraviolet (UV) spectral range, providing compatibility with CMOS process technology.^7,16–18^ Bismuth nanodisk arrays deposited by pulsed laser deposition have also emerged as a UV plasmonic material.^6^ An unconventional plasmonic metal that has not been explored widely is indium,^19^ which has a plasmon resonance in the near-UV visible spectral range.^16^ Previously, indium nanoparticle arrays were formed by evaporation on a silicon substrate, followed by dewetting, resulting in small grains that do not coalesce.^20,21^ The optical properties of these geometries have been characterized by transmittance and reflectance spectroscopy.^20,22,23^ Colloidal indium nanostructures with a sub-10 nm diameter have also been synthesized. These particles showed unusual redshift in absorption with decreasing particle size, attributed to ligand and confinement effects.^24^ Furthermore, due to oxidation, a 3 nm oxide shell forms around the particles,^25^ for decreasing particle sizes (<30 nm), this has been shown to quench the LSP resonance.^26^ Finally, indium arrays have been made by combining substrate conformal imprint lithography (SCIL) with electrodeposition, offering a means of producing scalable plasmonic lattices with a strong plasmonic LSP resonance due to enhanced in-plane light scattering.^27^

In this paper, we present a method to selectively deposit indium with a high optical quality. Similar to selective area epitaxy used for the deposition of III–V nanostructures,^28^ the process includes the patterning of silicon (Si) substrates with predefined widths, pitches, and lengths. Both inverted pyramids and V-groove geometries were made by chemical etching in silicon. Subsequently, indium is deposited by using molecular beam epitaxy (MBE), ensuring high purity. Atomic-force microscopy (AFM) and scanning electron microscopy (SEM) measurements are performed to characterize the morphology of the indium droplets after deposition. Under our deposition conditions, indium fully wets the V-grooves. This wetting behavior results from confinement provided by the patterned substrate combined with the optimization of surface energy, resulting in nanostructure arrays with smooth and flat surfaces. We characterize the optical properties of the indium arrays with cathodoluminescence (CL) spectroscopy, which provides a high spatial imaging resolution. Individual indium structures show a clear LSP resonance that shifts with an increasing particle size. Indium nanowires, grown inside V-grooves, we find clear CL interference characteristics for the generation of SPPs, and the angular distribution of the CL emission clearly shows the interference due to the square periodicity of the structure. Overall, our findings suggest that indium deposition by MBE enables the fabrication of well-defined, uniform, and reproducible metal nanostructure arrays that can find use in optical applications in the ultraviolet spectral range.

## Results and Discussion

### Indium Deposition

A Si (100) substrate is covered with an 18 nm thick SiO_2_ mask featuring diverse patterns of varying widths, pitches, and lengths. Our focus lies on two different shapes: inverted pyramids and V-grooves aligned along <110> directions (illustrated in Figures 1a and 2a, respectively). Along these directions, the trenches have a 111 family of side facets resulting from directional KOH etching. These facets are not fully formed for inverted pyramids with larger widths for which a flat (100) bottom is present. The presence or absence of flat (100) surfaces at the bottom within the studied width range did not yield significant differences in the deposition of the observed structures.

**Figure 1:**
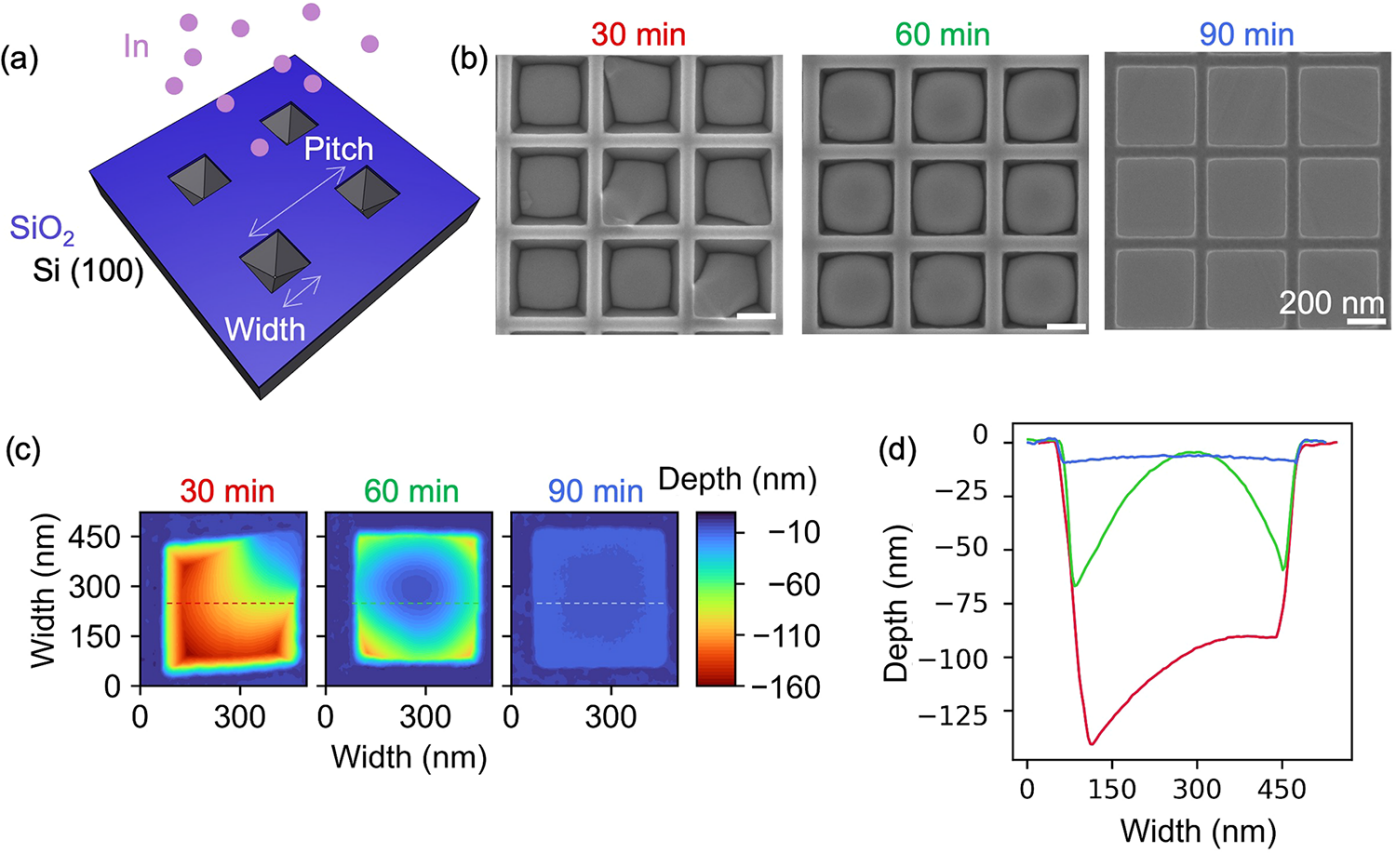
(a) Schematic showing the etched substrate covered with oxide and indium deposition within inverted pyramids; (b) SEM images of temporal evolution of indium deposition for 30, 60, and 90 min (scale bars are 200 nm); (c) corresponding AFM images of single structures shown in (b); and (d) line scans taken at the dashed lines shown in (c). The structures have a nominal width of 140 nm and a pitch of 500 nm.

**Figure 2:**
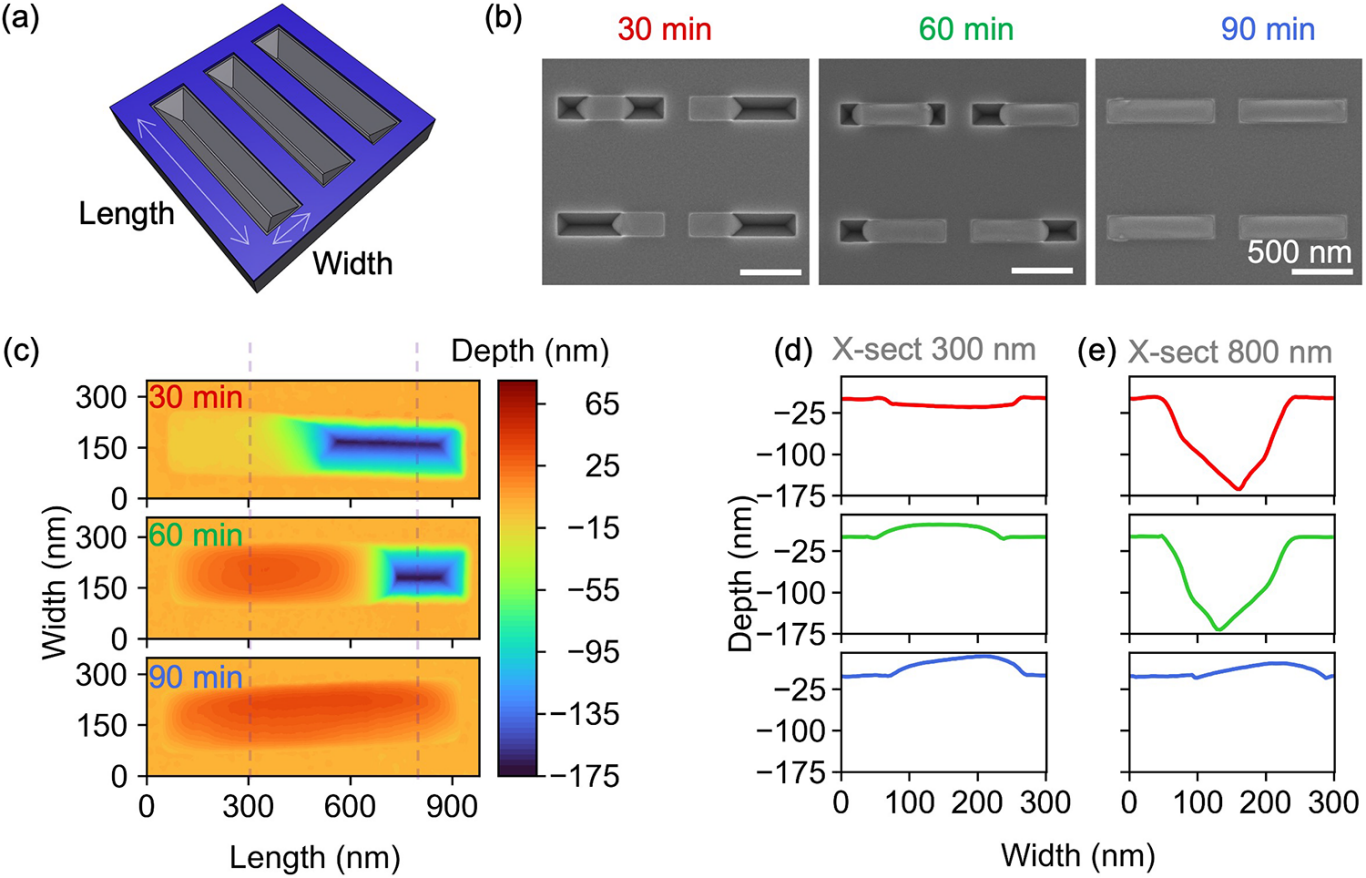
(a) Schematic of the substrate showing the width and length of a groove; (b) SEM images of temporal evolution of indium deposition for 30, 60, and 90 min (the scale bars are 500 nm); (c) AFM contour plots of one of the representative grooves shown in (b); and (d, e) line scans taken from the dashed lines shown in (c) at lengths of 300 and 800 nm, respectively.

We varied the deposition temperature between 500, 550, and 600 °C to find an optimized selective deposition temperature (see SI, Figure S1). At 500 °C, the growth was not selective, and indium droplets covered the substrate. Above 550 °C, we started to observe selectivity, and for the rest of the experiments, we conducted our depositions at 600 °C. We did not investigate the upper temperature limit of indium deposition, as this would require a systematic study quantifying the deposited volume within the structures. Furthermore, having an oxide-free Si surface and an ultrahigh vacuum environment of MBE enhances the wetting of indium metal.

Figure 1 shows the selective indium metal deposition process within inverted pyramids. In Figure 1b, SEM images depict the temporal evolution of the incorporation of indium within inverted pyramids subjected to deposition times of 30, 60, and 90 min. We clearly see that the deposition is selective, with no crystallites on the mask region. Longer deposition times result in a higher liquid indium volume within the holes. To study the temporal depth evolution, Figure 1c presents the 3D AFM contour plots of the individual structures for deposition times of 30, 60, and 90 min. Indium predominantly wets the bottom; however, in some grooves, metal pinning by the mask occurs at the edge of the SiO_2_. By 90 min, the liquid reaches almost the surface level for this width. Horizontal line scans along the width direction, as indicated by dashed lines in Figure 1c, are shown in Figure 1d. Until the pyramid volume is filled, the contact angle of the liquid decreases, resulting in a flat surface morphology that thoroughly wets the surface. Within inverted pyramids, initial droplet formation occurs either at the bottom of the structures or at the interface between oxide and Si surfaces. There is no notable difference in filling amount among the various pitches analyzed for inverted pyramids; however, these structures exhibit similar heights, indicating no pitch dependency (see Supporting Information, Figure S2a).

Next, we studied the indium deposition in the V-grooves. Figure 2a shows the schematic of the deposition process, and Figure 2b exemplifies the temporal evolution of the incorporation of indium within the V-grooves. Height information is depicted in the 3D contour plots captured by AFM in Figure 2c, where the top-to-bottom progression corresponds to 30, 60, and 90 min deposition intervals. Figure 2d,e shows the cross sections taken from the dashed lines at 300 and 800 nm in Figure 2c. As the deposition time increases, metal fills the entire groove, nearly filling it up. Within V-grooves, the initial droplet formation occurs at one of the two side corners, with it occasionally commencing in the middle of the groove. As the V-groove length increases, the probability of indium droplets forming at multiple locations increases. Furthermore, at a given time, increasing lengths result in height variations for a constant width (Figure S2b). Under optimized deposition conditions, once droplets form, instead of swelling out, they form a horizontal metal nanowire with a smooth top surface. This indicates that as the deposition time increases, incoming indium adatoms preferably incorporate an inclined growth front rather than at the top facet. After reaching a certain volume with an increasing deposition time, the droplet contact angle increases, extending beyond the mask. After certain conformal filling, an increase in volume results in a perturbation in droplet stability, which causes droplet swelling, collecting more indium adatoms, and acting as a sink (see Figure S2c). This behavior is easily observed in smaller-width trenches at 90 min of deposition due to the relatively low volume of the trench (see Figure S3).

The equilibrium wetting behavior of indium droplets inside the trenches was modeled using Surface Evolver, assuming a uniform contact angle of 72° on all surfaces (see Supporting Information, Section S5). Two initial configurations were simulated: a “one-sided” droplet at a terminating face and a “two-sided” droplet positioned away from both faces. As the droplet volume increased, it advanced along the trench while maintaining nearly constant mean curvature, with distinct transitions depending on trench length—either continuous advancement or division and later coalescence. After the triple line became fully pinned at the trench’s top edges, the droplet evolved by forming a bulge with an increasing apparent contact angle until depinning occurred. In V-groove trenches, a similar evolution was observed, but the droplet advanced more rapidly due to the geometry of the sidewalls.

### Optical Properties

To study the optical properties of the grown indium nanostructures, we used cathodoluminescence (CL) spectroscopy with a 30 keV electron-beam energy. By scanning the electron beam over the sample with a pixel size of 10 nm, we built up a CL excitation map for the indium geometries.

Figure 3a shows the CL spectra for spherical indium nanoparticles with diameters of 85 (blue), 100 (orange), 110 (green), and 150 nm (red), excited by the electron beam at the edge of the particle. The inset displays the SEM image of the smallest particle. Figure 3b shows the scattering efficiency for the indium particles calculated using Mie theory and the dielectric constants from the reference Palik.^29^ The scattering spectrum exhibits long-wavelength resonant electric dipole (ED) and shorter-wavelength quadrupole (EQ) modes. Note that for very small particles, Ohmic dissipation increases due to surface scattering effects; yet the present work focuses on larger particles.

**Figure 3:**
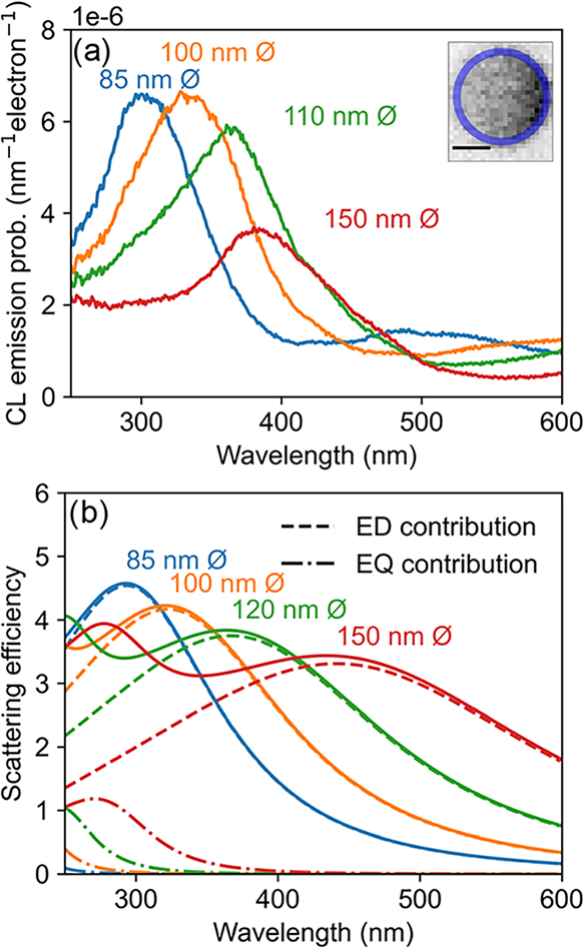
(a) Measured CL spectra for spherical indium nanoparticles on a Si substrate covered with an 18 nm SiO_2_ layer, with diameters of 85 nm (blue), 100 nm (orange), 110 nm (green), and 150 nm (red). The inset shows an SEM image of the 85 nm-diameter particle. The scale bar is 50 nm. The nanospheres are excited by 30 keV electrons, and the CL is integrated over the pixels at the edge of the particle, indicated by the blue circle in the inset. (b) The scattering cross-section was calculated with Mie theory and normalized to the geometrical cross-section for spherical indium nanoparticles in vacuum with the same diameter as in (a). The contributions of electric dipole (ED) and quadrupole (EQ) resonant modes are indicated by dashed lines.

Comparing the measurements and the calculations in Figure 3, we see similar trends. The peak wavelength (300 nm) and line width (70 nm FWHM) for the 85 nm-diameter particle agree well between measurement and simulation. The EQ mode seen in the simulations is not observed in the experiments. Our earlier work has shown that the modal excitation efficiency of plasmonic nanoparticles depends strongly on the electron position on the particle, as that determines the phase-matching condition at which efficient CL excitation occurs.^30^ At the particle edge, the electron beam couples mostly to the ED mode, and hence, the EQ mode is not visible in the CL spectrum. Furthermore, we find that both experiments and simulations show a redshift with increasing particle size, as expected due to weaker restoring forces on the plasmonic charge displacement at larger sizes. Discrepancies between the two may be due to differences in the optical constants of indium for the experimental particles compared with the literature values used. Additional data for excitation at the particle center and corresponding boundary element method calculations to calculate CL emission are shown in the Supporting Information (Figure S6).

Next, we studied the SPP modes of the indium structure within the V-grooves. Figure 4a shows the SEM image of indium deposited inside a V-groove. Note that the indium deposition is noncontinuous within the V-groove. We scanned the electron beam from the top to the bottom with a step size of 10 nm, and the CL spectrum was collected at every pixel. This results in the CL map of Figure 4b that shows a spectrum for every pixel on the *y*-axis corresponding to the red line in Figure 4a. In the data, we clearly see minima and maxima due to the destructive and constructive interference of SPP modes launched by the electron beam and reflected on the edge of the particle. The period of the standing waves is expected to match half the SPP wavelength given by^31^

1 $$\lambda_{\mathrm{SPP}}=\lambda_{0}/n_{\mathrm{eff}}$$

where the effective refractive index (*n*_eff_) is given by^31^

2 $$n_{\mathrm{eff}}=\sqrt{\epsilon_{d}\epsilon_{m}/\left(\epsilon_{d}+\epsilon_{m}\right)}$$

Overlaying the expected spatial period with the measurements (dashed white line), we find good agreement. At a wavelength of 350 nm, the calculated SPP wavelength is 330 nm. In the experimental data, the average distance between the minima was found to be 160 nm at a wavelength of 350 nm (see Supporting Information, Figure S7 for a linescan at 350 nm wavelength), corresponding to half the SPP wavelength. The propagation length of the SPP is given by^32^

$$\delta_{SPP}=\frac{\lambda_{0}}{2\pi }\left(\frac{\epsilon_{m\prime }^{2}}{\epsilon_{m}^{\prime }}\right){\left(\frac{\epsilon_{m}^{\prime }+\epsilon_{d}}{\epsilon_{m}^{\prime }\epsilon_{d}}\right)}^{3/2}$$ 3

At a wavelength of 350 nm, the calculated SPP propagation length at a planar indium-air interface is 1500 nm. In the experimental data, we see two periods in the CL map at 350 nm wavelength, and the visibility decreases toward the center of the wire. We estimate the propagation length to be two periods, corresponding to 640 nm, which is twice the SPP wavelength. This indicates that the grown indium has somewhat higher losses than expected based on the literature, in agreement with the larger FWHMs observed in the experiments shown in Figure 3a. Coupling of SPPs across the indium nanowire surface can also reduce the SPP propagation length.

**Figure 4:**
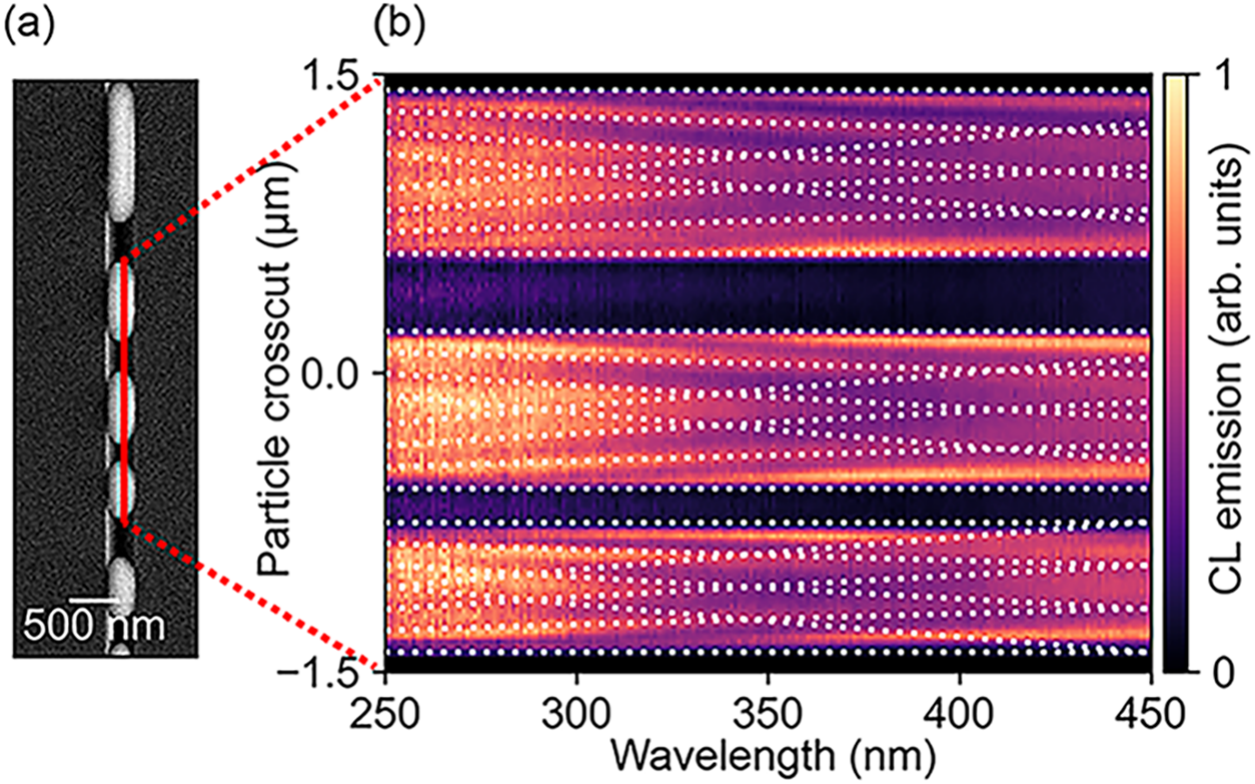
(a) SEM map showing the indium structure within V-grooves and (b) corresponding CL spectra along the line indicated in (a). The dashed white lines show the expected maxima of standing SPP waves with a period of half the wavelength of the SPP mode.

Finally, we investigate the angular distribution of the CL emission of square indium arrays embedded inside inverted pyramids. Angle-resolved CL measurements are performed using a 70 nm bandwidth filter centered at a wavelength of 400 nm. The angular emission is represented in reciprocal-space coordinates, where *k_x_* and *k_y_* denote the in-plane components along the *x*- and *y*-axis, normalized to the free-space wavevector (*k*_0_ = 2π/λ). Figure 5a–c shows the result for an indium array with a pitch of 2000 nm. Figure 5a,b shows an SEM image of the structure and the corresponding angle-resolved CL intensity, measured with electron beam excitation at the center of the structure (indicated by the red dot in Figure 5a). Figure 5c shows the calculated emission profile for this geometry using rigorous coupled-wave analysis (RCWA), which models the electromagnetic wave diffraction through a periodic structure.^33^ The simulation calculates the absorption of the array when it is illuminated with a plane wave at varying angles. Due to reciprocity, the absorption profile is analogous to the emission pattern. While we do not expect a perfect correspondence between the RCWA model and the CL emission—since CL involves localized, point-source excitation—the diffraction features (i.e., angular grating conditions) are expected to be similar. The primary difference lies in the amplitude, which reflects the coupling efficiency to the optical modes. Unlike a plane wave, which contains both s- and p-polarized components, an electron beam predominantly excites modes, emitting p-polarized light. Comparing Figure 5b,c, we observe the same trend of strong emission at the high angles. This is due to the *z*-oriented dipole excited at the center of the structure. Due to the large pitch, the diffraction bands are only visible at very high emission angles in the simulations. In the measurements, the angular resolution is insufficient to resolve them.

**Figure 5:**
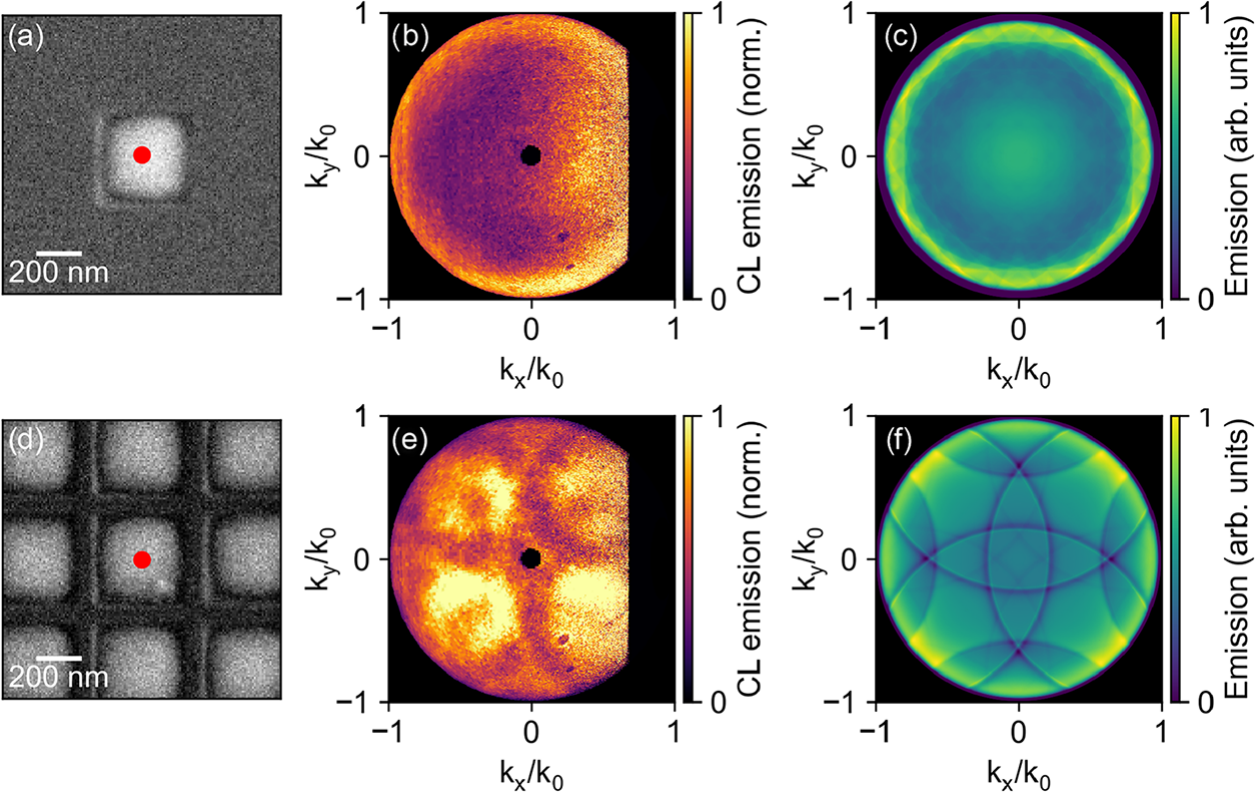
(a) SEM image of the indium-infilled array of inverted pyramids with a width of 370 nm and a pitch of 2000 nm, showing the electron excitation position (red dot). (b) Experimentally measured angle-resolved CL emission intensity at a wavelength of 400 nm, and (c) simulated emission profile obtained from RCWA. (d–f) Corresponding results for a similar array with a pitch of 500 nm. The emission arrays are plotted as a function of the normalized in-plane wavevector along the *x*- (*k*_*x*_/*k*_0_) and *y*-direction (*k*_*y*_/*k*_0_), where *k*_0_ = 2π/λ.

For comparison, we also study an array with a 500 nm pitch, shown in Figure 5d–f. Figure 5d shows the SEM image of the structure, while Figure 5e,f represents the angle-resolved CL measurement (again with center excitation, marked by the red dot) and the corresponding RCWA simulation, respectively. The angle-resolved CL emission distribution clearly shows a far-field interference pattern characteristic of square symmetry of the lattice. Comparing the measurements to the simulations, we clearly see similar dark diffraction bands, where the light cannot couple to the far-field. Furthermore, the intensity is homogeneously distributed across the upper hemisphere, in contrast to the large-pitch structure, which radiated more strongly at high emission angles. Additional data for arrays with intermediate pitches (750 and 1000 nm) are provided in the Supporting Information (Figure S8) and show how the diffraction bands move depending on the pitch of the lattice.

## Conclusion

In conclusion, we have presented a process sequence for the selective deposition of indium on patterned Si(100) substrates. Both inverted pyramid structures and V-grooves form effective templates for the formation of indium nanostructures. The indium filling and wetting behavior depend on the pattern length-to-width ratio. Cathodoluminescence spectroscopy on the indium nanoparticles shows a strong electric dipole plasmon resonance, indicating a good optical quality of the grown indium material. Indium nanowire structures show SPPs with standing waves due to interference, showing sizable SPP propagation lengths along the wires. Overall, our findings highlight the efficacy of MBE in creating controlled indium nanostructures and arrays with high optical quality in the ultraviolet spectral range.

## Experimental Methods

### Substrate Fabrication

Intrinsic Si (100) wafers served as substrates for indium deposition. A 25 nm thick thermal SiO_2_ mask layer was grown on the as-received wafers to achieve selective deposition. The desired pattern was created employing electron-beam lithography (EBL) on a ZEP positive resist and cold development with n-amyl acetate. Following the transfer of the desired pattern to the mask via reactive ion etching (RIE) with CHF_3_/SF_6_, the polymeric resist was removed using a high-power oxygen plasma. Subsequently, the native oxide was removed with a 1% HF solution for 20 s, and the wafer was immersed for 8 min in a 40% KOH solution to form the V-grooves within the mask openings. The resulting wafer was cleaved into 1 cm^2^ chips. Note that there is a difference between nominal and actual width values due to the fabrication process, including EBL patterning and subsequent etching. Unless stated otherwise, the values given will be the nominal ones.

### MBE Deposition

After a final immersion in a 1% HF solution for 1 min to remove any remaining native oxide, the chips were immediately introduced into the ultrahigh vacuum environment of a Veeco GENxplor MBE system. After the predegassing step at 400 °C for 2 h, samples are transferred into the growth chamber. Annealing is performed at a temperature of 800 °C for 30 min. Then, the temperature is decreased to the indium deposition temperature. The indium beam equivalent pressure (BEP) is 3.6 × 10^–7^ Torr, which is enough to achieve good selectivity, and the deposition time is 60 min. After that, the substrate is cooled to 100 °C. Note that the temperatures reported are manipulator-set temperatures.

### Cathodoluminescence Measurements

CL measurements were performed using a FEI Quanta FEB 650 SEM (Thermo Fisher Scientific Inc., MA) and a SPARC Spectral CL collection system (DELMIC BV, The Netherlands).^34^ The measurements used 30 keV electrons at a beam current of 2.3 nA. For the spectral measurements, an exposure time of 1 s was used at two center wavelength settings of the grating (350 and 500 nm). For every spherical particle, a map was made of the entire particle and the signal was integrated over the pixels at the edge of the particle. To correct for the background, a spectrum taken with the electron beam off was subtracted, and the total signal was normalized by using the system response function.

For the angle-resolved CL measurements, we directly imaged the light emanating from the sample through the parabolic collection mirror onto an imaging camera and correlated each pixel on the camera to an azimuthal and radial emission angle from the sample. The angular emission for the inverted pyramids was measured by using a 400 nm filter with a bandwidth of 70 nm and a 5 s exposure time, while the electron beam was placed at the center of one of the structures. The measured size of the inverted pyramids was 370 nm, and the pitches were 500, 750, 1000, and 2000 nm.

### RCWA Simulations

The angle-resolved emission intensities were computed using rigorous coupled-wave analysis (RCWA) simulations from an open-source software package S^4^: the Stanford Stratified Structure Solver by Liu and Fan.^33^ RCWA simulations compute the absorption in a periodic system for incoming plane waves under different angles, ϕ and θ. According to the reciprocity principle, the computed angle-resolved absorption profiles equal the emission profile at the same wavelength from the absorbing material, in this case, the indium within inverted pyramids. We perform the simulation at a wavelength of 400 nm for an indium array with a width of 370 nm (nominally 240 nm), embedded in a Si substrate with 18 nm of SiO_2_ on top. For all three materials, we use the dielectric constants from Palik.^29^

## Supplementary Material





## Notes

Dependence of the indium deposition on temperature (Figure S1); pitch, length, and width (Figures S2 and S3); AFM and SEM images of unfilled structure (Figure S4); modeling the indium deposition (Figure S5); additionally, to further elaborate on the optical characterization, there are additional figures: CL spectra for spherical particles for both edge and center excitation (Figure S6); observation of SPP waves within V-grooves (Figure S7); and the angular distribution of CL emission for a square lattice of indium structures depending on the pitch of the lattice (Figure S8) (PDF)
Wetting behavior (Videos S1, S2, and S3) (ZIP)
