# Supplementary material for: Selective-Area Deposition of Indium and Its Plasmonic Properties
Source: ACS Appl Opt Mater. 2025 Dec 10;3(12):2826–32. doi: 10.1021/acsaom.5c00373 (PMC12751113; doi:10.1021/acsaom.5c00373)
Supplement: Supplementary file 1 [file ot5c00373_si_001.pdf]

Supporting Information

Selective-Area Deposition of Indium and its Plasmonic Properties

Didem Dede,[†,‡] Evelijn Akerboom,[†,¶] Riccardo Brondolin,[‡] Tom Veeken,[¶] Thomas Hagger,[‡] Raphael Lemerle,[‡] Esther Alarcon Llado,[¶] Valerio Piazza,[‡] W. Craig Carter,[§] Albert Polman,[*,¶] and Anna Fontcuberta i Morral[*,‡,‖]

†*Equal contribution*

‡*Laboratory of Semiconductor Materials, Institute of Materials, École polytechnique fédérale de Lausanne, Route Cantonale, Lausanne, Vaud 1015, Switzerland*

¶*Center for Nanophotonics, NWO-Institute AMOLF, 1098XG Amsterdam, The Netherlands*

§*Department of Materials Science and Engineering, Massachusetts Institute of Technology, Cambridge, Massachusetts, 02139, United States*

‖*Institute of Physics, École polytechnique fédérale de Lausanne, Route Cantonale, Lausanne, Vaud 1015, Switzerland*

E-mail: a.polman@amolf.nl; anna.fontcuberta@epfl.ch

S1: Indium deposition dependence on temperature

Figures S1 (a-c) show the dependence of the indium deposition at different temperatures within different structures on the mask. At 500°C, multiple droplets on the mask can be observed. At 550°C, there is a significant improvement in the selectivity. There are very few droplets on the mask. At 600°C, there are almost no droplets. In (d), we showed some additional zoomed-out images from the deposition at 600°C, and marked the few droplets on the mask with arrows.

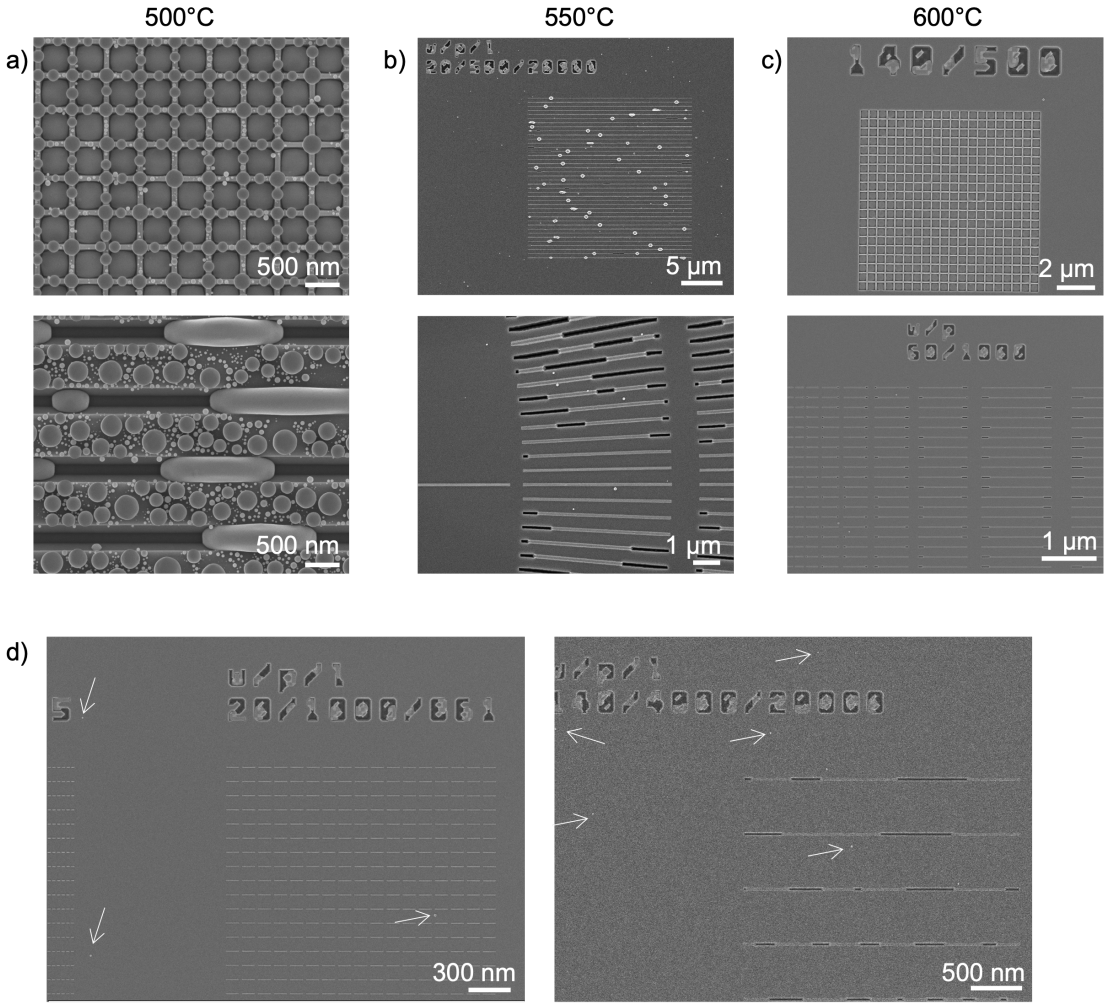

Figure S1: The deposition at different temperatures (a)500°C, (b)550°C, and (c)600°C. (d) Deposition at 600°C showing some small particles on the mask.

S2: Indium deposition dependence on pitch, length, and width

Figures S2a and b depict the dependence of indium deposition at a certain time for inverted pyramids with different pitches and V-grooves with different lengths, respectively. Figure S2c shows the temporal evolution of the filling inverted pyramid structures and compares it to the V-grooves with different widths.

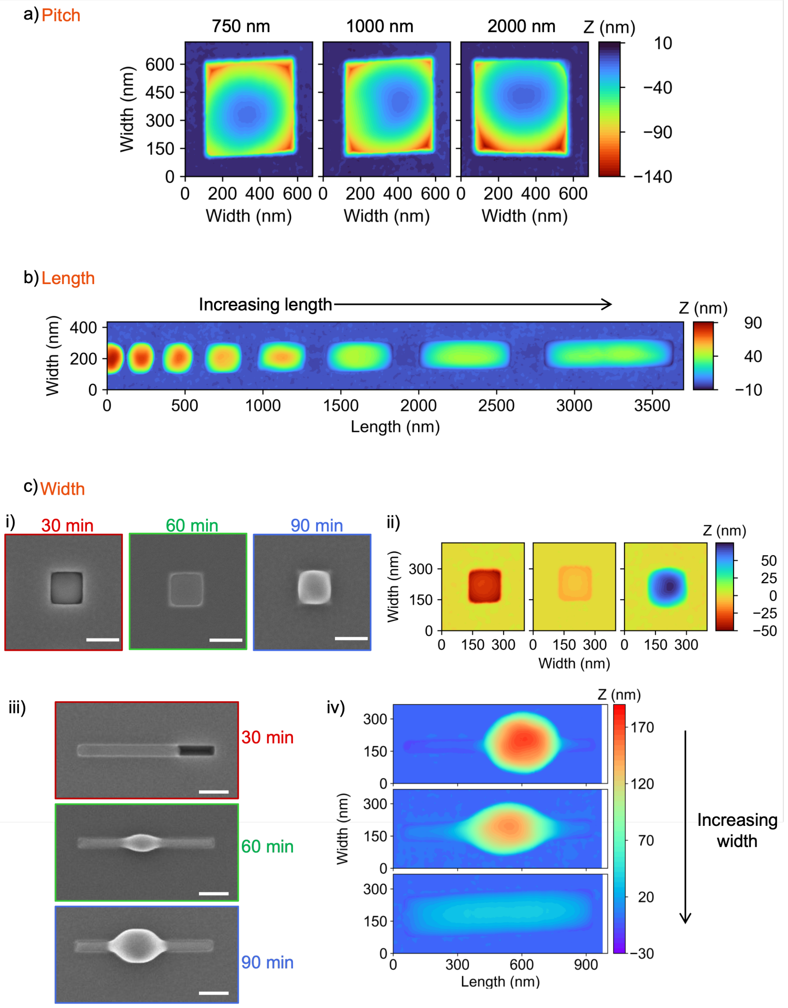

Figure S2: The deposition in different pitches, lengths, and widths. AFM maps of (a) indium particles in inverted pyramids with different pitches and (b) grown in V-grooves with different lengths. (c) Time evolution of the indium growth in inverted pyramids and in V-grooves. (i), and (iii) SEM images for indium particles in inverted pyramids deposited at different times (c) i and corresponding AFM maps of the same structures (c) ii. SEM images for indium structure in V-grooves deposited at different times (c) iii and corresponding AFM maps of the same structures (c) iv. All the scale bars are 200 nm.

S3: Growth dependence on V-groove trench width

Figure S3 shows the resulting indium structures after 90 minutes of deposition in the V-grooves with different nominal widths: 20 nm, 40 nm, and 140 nm, from left to right. Smaller trenches result in more droplet swelling outside of the trench.

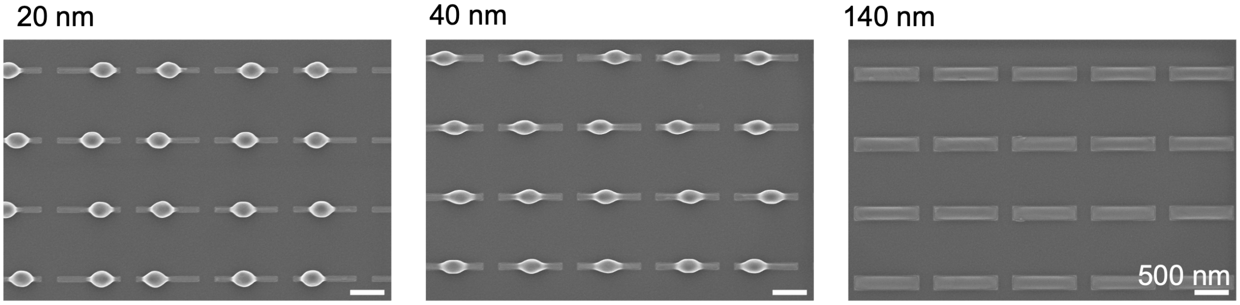

Figure S3: SEM images for final indium structures deposited for 90 minutes in V-grooves with 1 μm pitch and different widths; 20 nm, 40 nm, and 140 nm, from left to right. Scale bars are 500 nm.

S4: AFM and SEM images of unfilled patterns

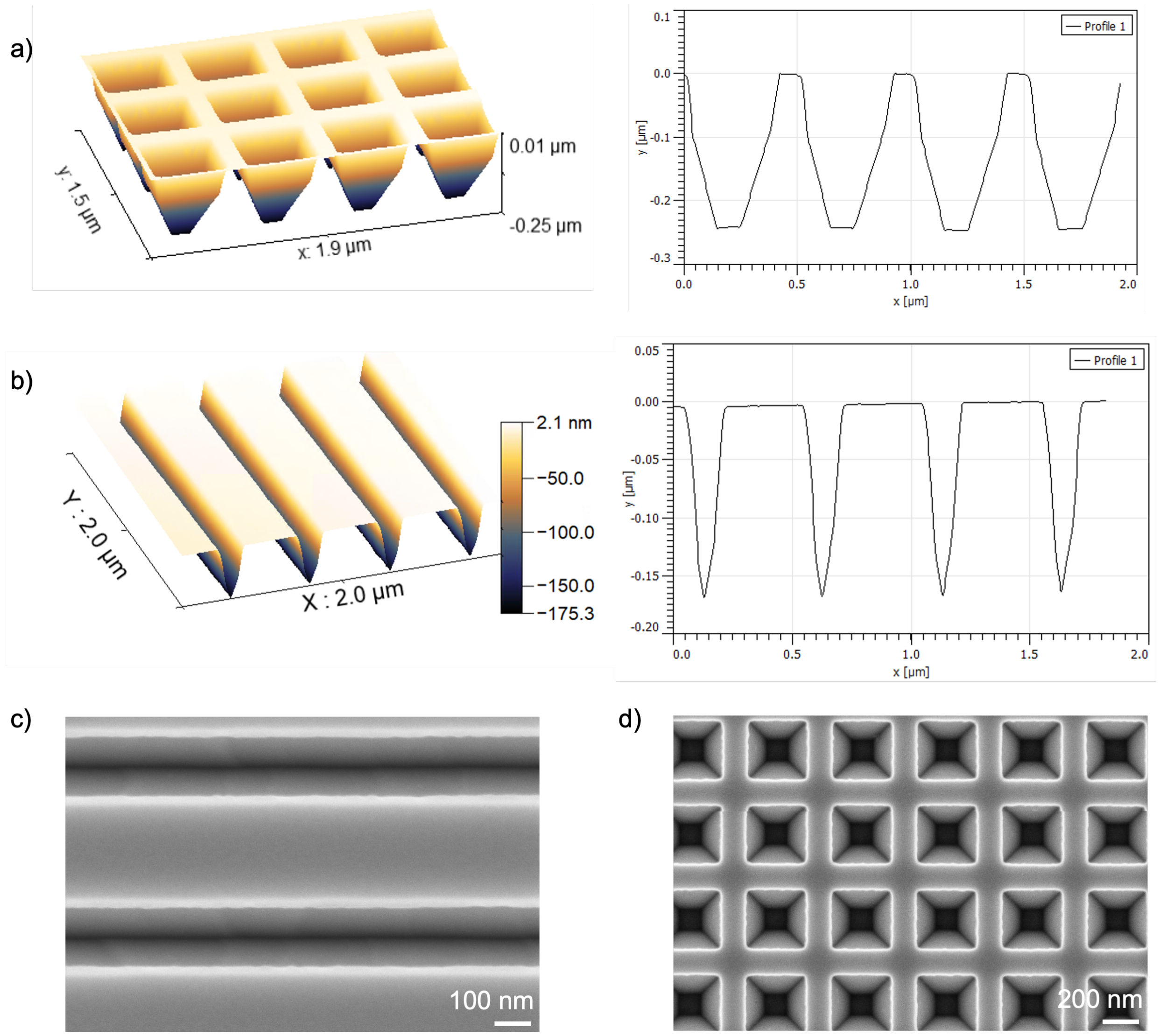

Figure S4: AFM and SEM images of structures without any indium deposition. AFM 3D image and the line profile of nominal (a) 140 nm width and 500 nm pitch inverted pyramids, (b)140 nm width, 500 nm pitch, and 20 μm length V-groove. (c) SEM image of structures given in (b). (d) SEM image of unfilled inverted pyramid with nominal 100 nm width and 500 nm pitch.

S5: Modelling the wetting behavior

It is assumed that indium only deposits onto an existing indium liquid droplet inside the rectangular trench. If the droplet can adjust its shape on a timescale that is rapid compared to deposition, its shape can be treated as an equilibrium capillary surface.

We performed computations of the equilibrium surface using Surface Evolver.[?] The indium droplet was modeled inside a rectangular trench with an aspect ratio (width/depth) of 1, assuming uniform wetting contact angle ϕ=72° on all substrate surfaces— bottom, side walls, and top. At the edges, where the droplet triple line is pinned, the contact angle may vary within a range constrained by the equilibrium angles of the adjoining surfaces. The droplet shape is further constrained by a mirror symmetry plane located at the center of the trench, parallel to the trench sidewalls, and orthogonal to the terminating side faces.

We simulated three growth modes by increasing the droplet volume for two initial configurations: (i) "one-sided", where the initial droplet occupies the bottom edge of one of the trench's terminating faces; and (ii) "two-sided", where the droplet is initially positioned away from both terminating faces.

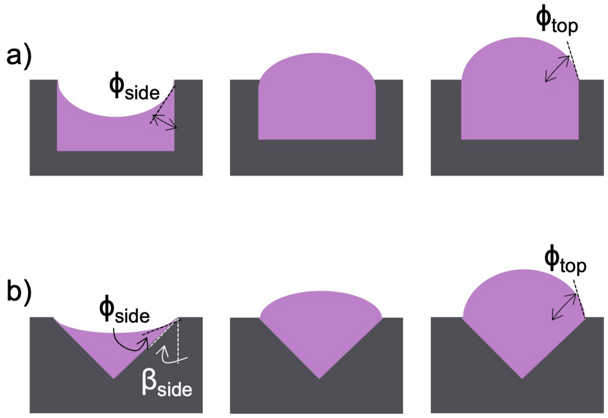

Figure S5: Cross-section images of the trenches showing the angles. Indium droplet deposited inside the a)rectangular trench, and b)V-groove.

In the "one-sided" case, shown in SI (Video S1), as the droplet volume increases, the liquid climbs along the terminating face until the triple line becomes pinned at the top edge. Further volume increase causes a droplet to advance into the trench by extending the pinned

triple line onto the top edge of the sidewalls and pushing a roughly self-similar protrusion along the trench axis. This protrusion has a triple line that descends along the sidewalls, crosses the bottom edge, and traverses the trough's bottom surface. During this propagation phase, the droplet surface maintains an approximately constant mean curvature, indicating a near-minimal energy configuration.

When the droplet reaches the opposite terminating face, its equilibrium shape undergoes an abrupt transition. While the triple line remains pinned to the top edges of the terminating faces, it retreats from the sidewall's edge towards the trench's bottom. The behavior following this transition depends on the trench length relative to its height, resulting in two distinct cases.

(1) Shorter trench case: If the trench length is short enough, the triple line remains pinned to the terminating surface's top edge, but the triple line abruptly retreats from the side's top edge towards the trough's bottom surface and maintains the equilibrium contact angle with the side surface (the determining factor between the two cases is whether there is enough volume at the transition so droplet surface remains above the bottom). As more volume is introduced, the triple line ascends until fully pinned to the trench's top edges.

(2) Longer trench case: For longer trenches, the droplet divides into two, each adhering to one of the terminating faces. These droplets continue to grow as isolated droplets until enough volume has been deposited that the two isolated protrusions collide and the isolated droplets coalesce. Once coalesced, the combined droplet evolves similarly to the short trench case, eventually advancing and pinning along the full trench perimeter.

In the "two-sided" case, shown in SI (Video S2), the protrusions advance along the trough until one of them collides with a side, after which the evolution proceeds as in the "one-sided" case.

After the droplet's triple line has been fully pinned at the trough's top edge, the droplet evolves by developing a bulge that is accommodated by an increasing "apparent" contact

angle (α) relative to the top surface. This angle can take on any value:

$$-\pi/2 + \phi_{contact-side} < \alpha < \phi_{contact-top} \tag{1}$$

where $\phi_{contact-side}$ and $\phi_{contact-top}$ are the equilibrium contact angles on the sidewalls and top surface, respectively. Once the apparent contact angle exceeds the top surface angle at any point on the triple line ($\alpha > \phi_{\text{contact-top}}$), the triple line will de-pin and the droplet will spread onto the top surface. This post-depinning spreading regime is not treated in this work.

Another potential instability arises when the bulge can redistribute its volume asymmetrically, the bulge becoming more pronounced at one end of the trench. While this instability has not been computed exactly, the mode is captured in the video given in the SI (Video S3).

For a V-groove trench with (111) side facets, the evolution is expected to follow a similar sequence to the rectangular case (Figure S5b). However, the rate at which the droplet advances with increasing volume is greater due to the changing geometry. In this case, the range of pinned contact angles would be

$$-\pi/2 + \phi_{contact-side} + \beta_{side} < \alpha < \phi_{contact-top} \tag{2}$$

where β_{side} is the side wall's angle with respect to the top surface.

S6: Optical characterization of spherical indium particles

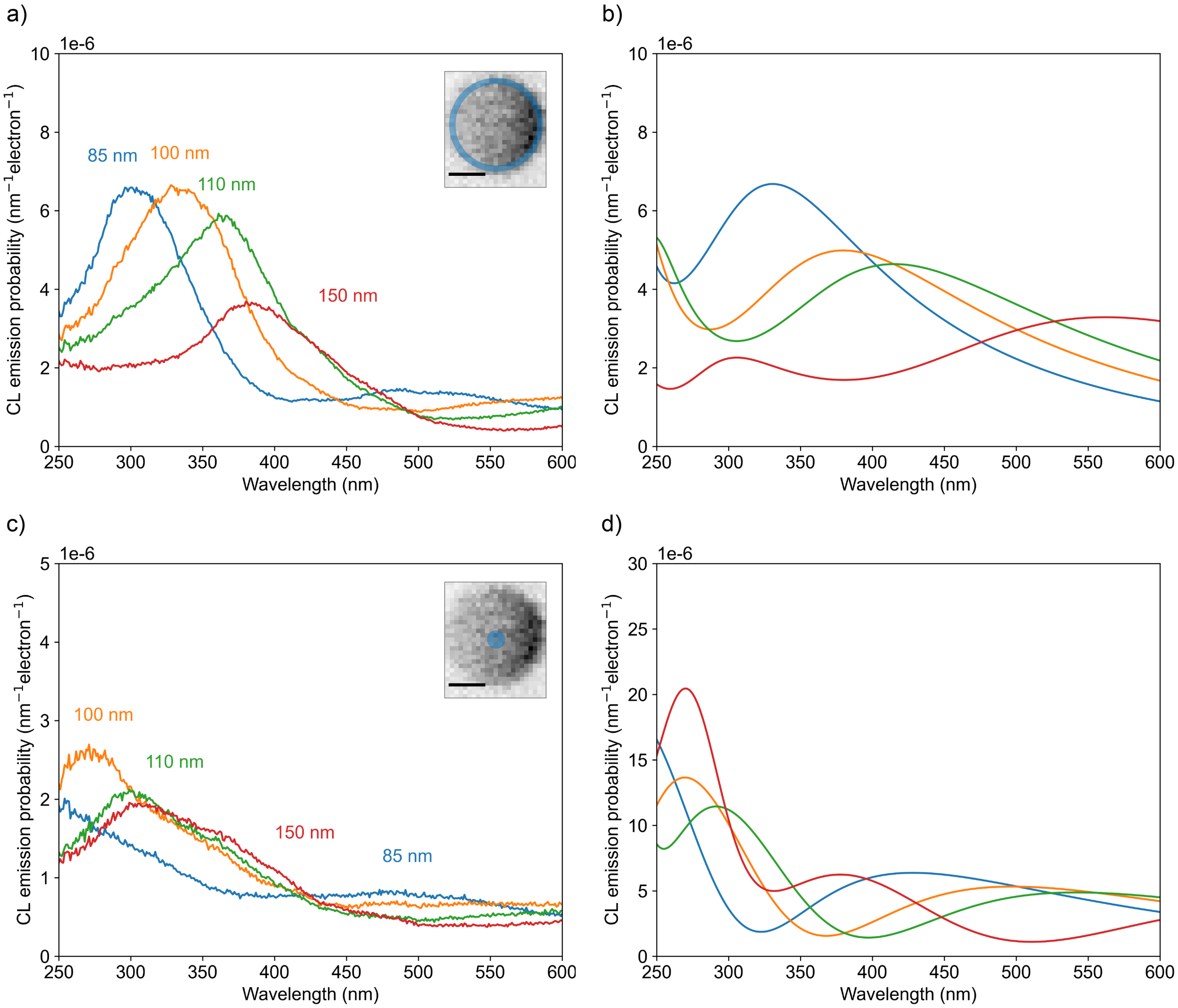

Figure S6: Measured CL spectra for spherical indium nanoparticles on a Si substrate covered with an 18 nm SiO_2 layer, with a diameter of 85 nm (blue), 100 nm (orange), 110 nm (green), and 150 nm (red). The inset shows an SEM image of the 85 nm particle (scale bar is 50 nm). The nanospheres are excited by 30 keV electrons, and the CL is integrated over (a) the pixels at the edge of the particle, indicated by the blue circle in the inset, and (c) integrated over the center pixels. CL spectra calculated using the boundary element method for indium nanoparticles excited (b) at an off-center position at 0.8 times the radius and (d) in the center of the particle, for the same particle sizes as in (a,c).

S7: The observation of SPP waves within V-grooves

To study the SPP waves on the In-air interface, we measure a CL map on In rods grown in the V-grooves. Figure S7a shows the SEM image of the In deposited inside a V-groove. We scanned the electron beam from the top to the bottom with a step size of 10 nm, and the CL spectrum was collected at every pixel. This results in the CL map of Figure S7b that shows a spectrum for every pixel on the y-axis corresponding to the red line in (a). The white dashed lines show the expected maxima of the CL, corresponding to the constructive interference of the SPP waves. To further examine the SPP wavelength, we take a crosscut of the CL map at a wavelength of 350 nm and show the CL intensity along the particle in Figure S7c. We clearly see minima, indicated by the black dashed lines with an average spacing of 160 nm, close to the expected value of 165 nm. In the crosscut, we see two periods, and the visibility decreases towards the center of the wire. We estimate the propagation length to be two periods, corresponding to 640 nm at 350 nm wavelength.

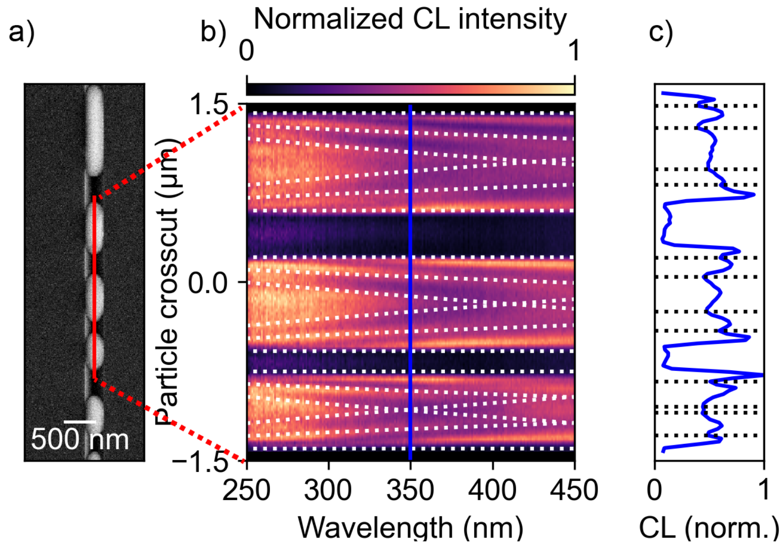

Figure S7: (a) SEM map showing indium structure within V-grooves and (b) corresponding CL spectra along the line indicated in (a). The dashed white lines show the expected maxima of standing SPP waves with a period of half the wavelength of the SPP mode. (c) Shows a crosscut of the CL intensity at a wavelength of 350 nm, indicated by the blue line in (b), with the minima indicated with black dashed lines.

S8: Angular distribution of CL emission for indium structures in a square lattice

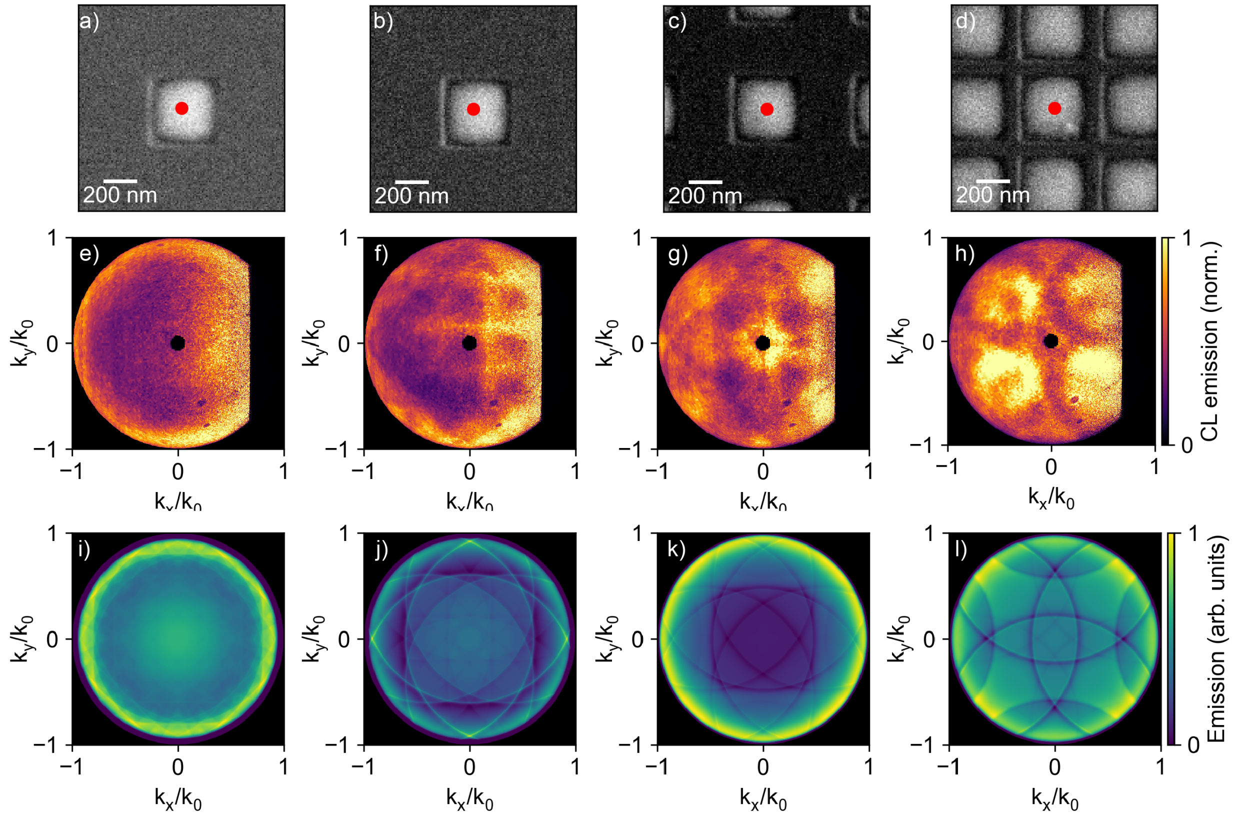

Figure S8: (a-d) SEM images of the indium structures within inverted pyramids with the electron excitation position shown as a red dot for an array with a pitch of 2000 nm, 1000 nm, 750 nm, and 500 nm, respectively (width 370 nm). (e-h) Corresponding experimental angle-resolved CL emission intensities measured with a 400±35 nm filter and (i-l) angle-resolved emission intensities from RCWA simulations for the particles in (a-d) calculated at 400-nm wavelength. The emission arrays are plotted as a function of the normalized in-plane wavevector along the x- (k_x/k_0) and y-direction (k_y/k_0), where $k_0 = 2\pi/\lambda$.
