# Supplementary figures and images for: Selective-Area Deposition of Indium and Its Plasmonic Properties
Source: ACS Appl Opt Mater. 2025 Dec 10;3(12):2826–32. doi: 10.1021/acsaom.5c00373 (PMC12751113; doi:10.1021/acsaom.5c00373)

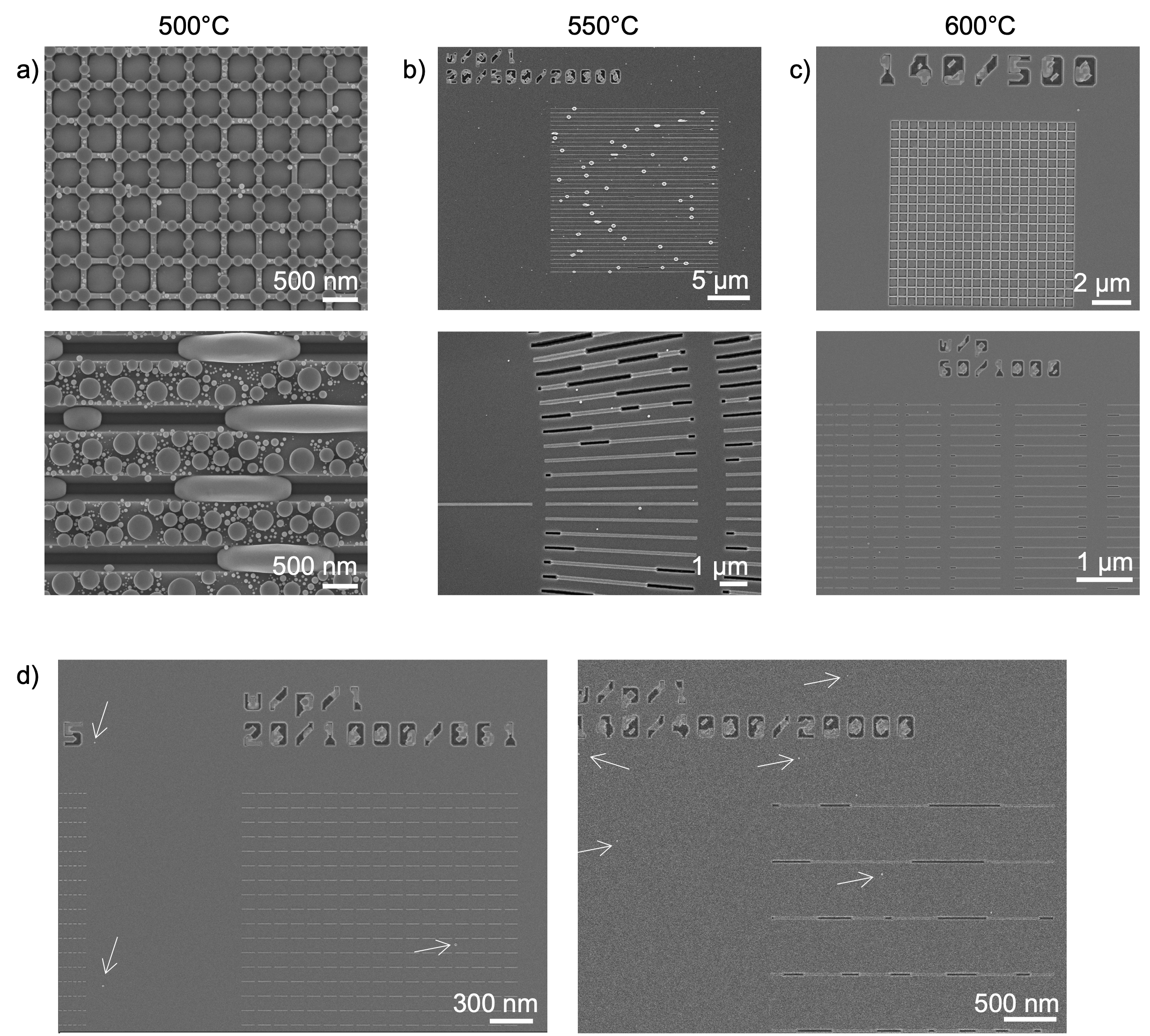

Supplement: Supplementary file 2 [file ot5c00373_si_002.zip › SI2 ot5c00373/SI/Images/FigureS1.jpg]

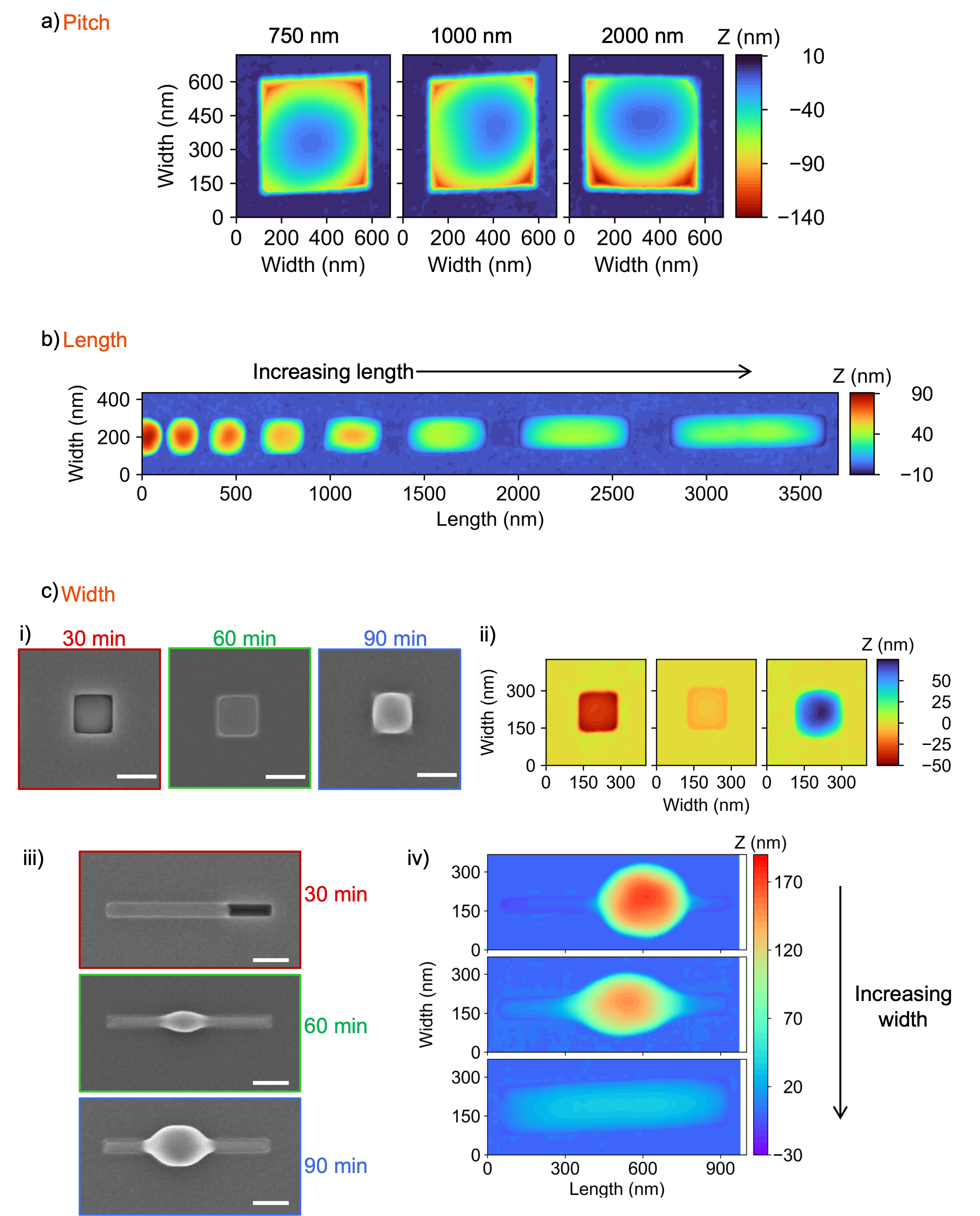

Supplement: Supplementary file 2 [file ot5c00373_si_002.zip › SI2 ot5c00373/SI/Images/FigureS2.png]

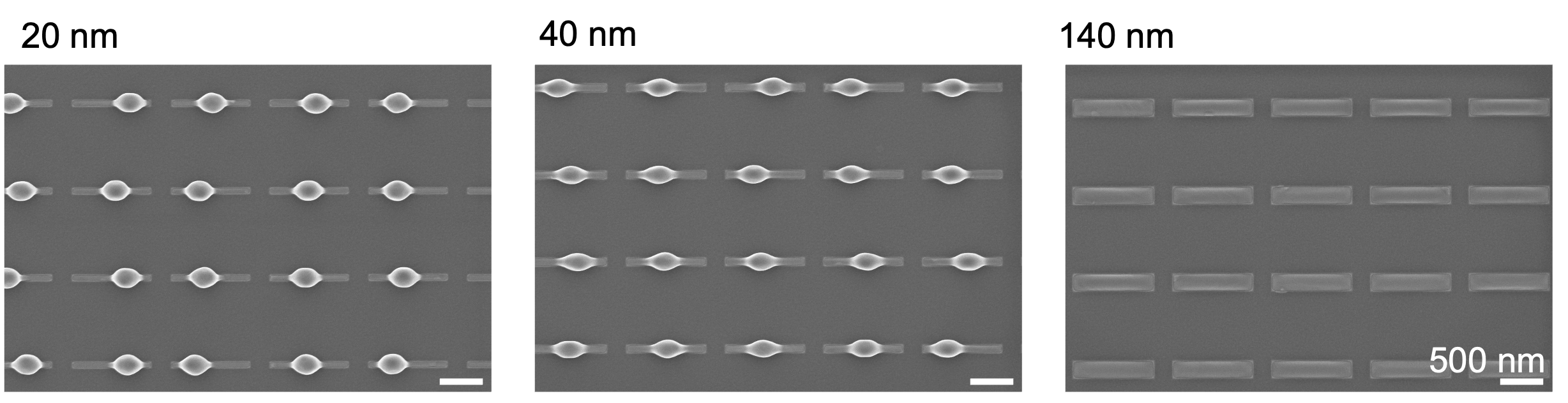

Supplement: Supplementary file 2 [file ot5c00373_si_002.zip › SI2 ot5c00373/SI/Images/FigureS3.png]

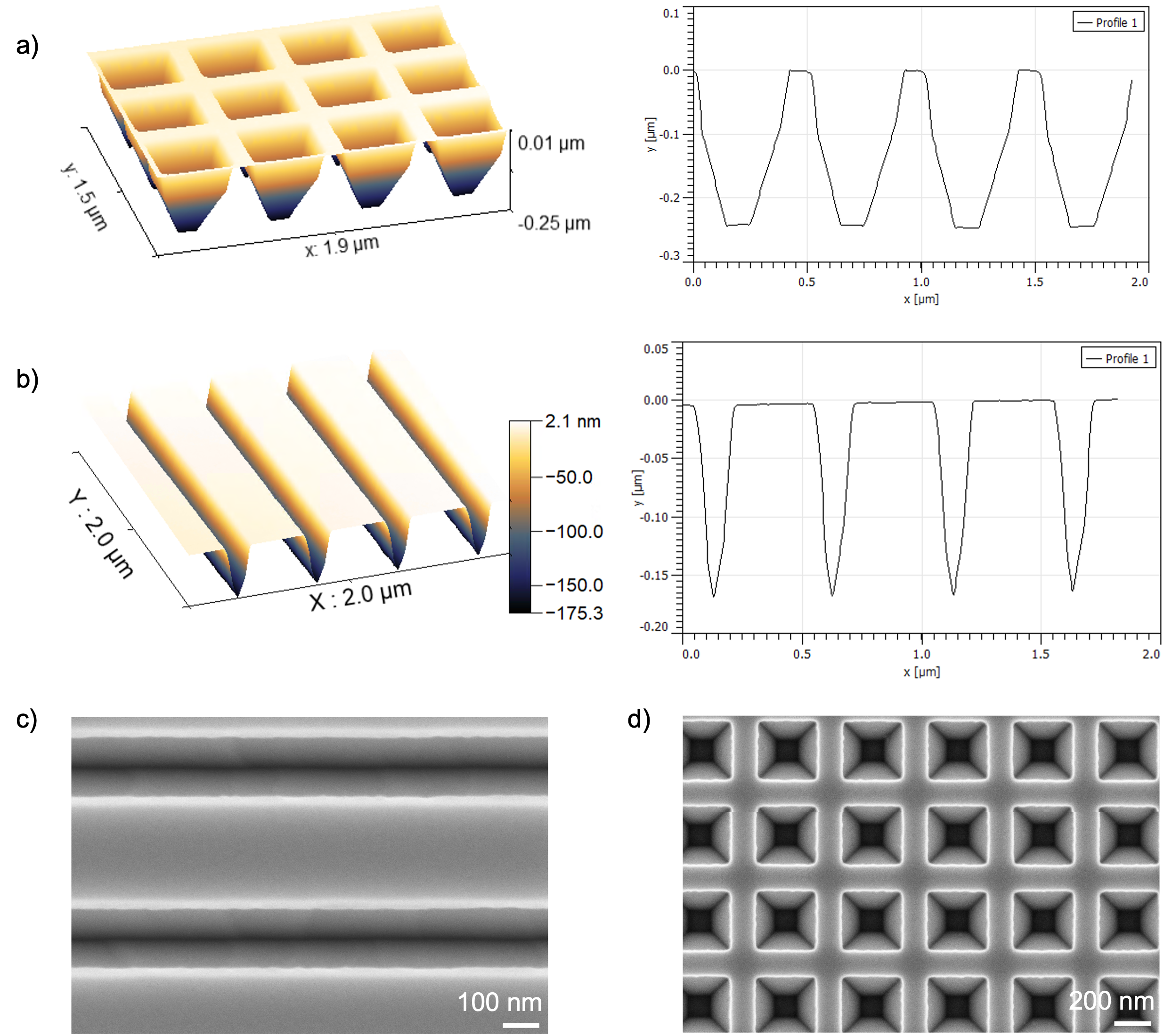

Supplement: Supplementary file 2 [file ot5c00373_si_002.zip › SI2 ot5c00373/SI/Images/FigureS4.png]

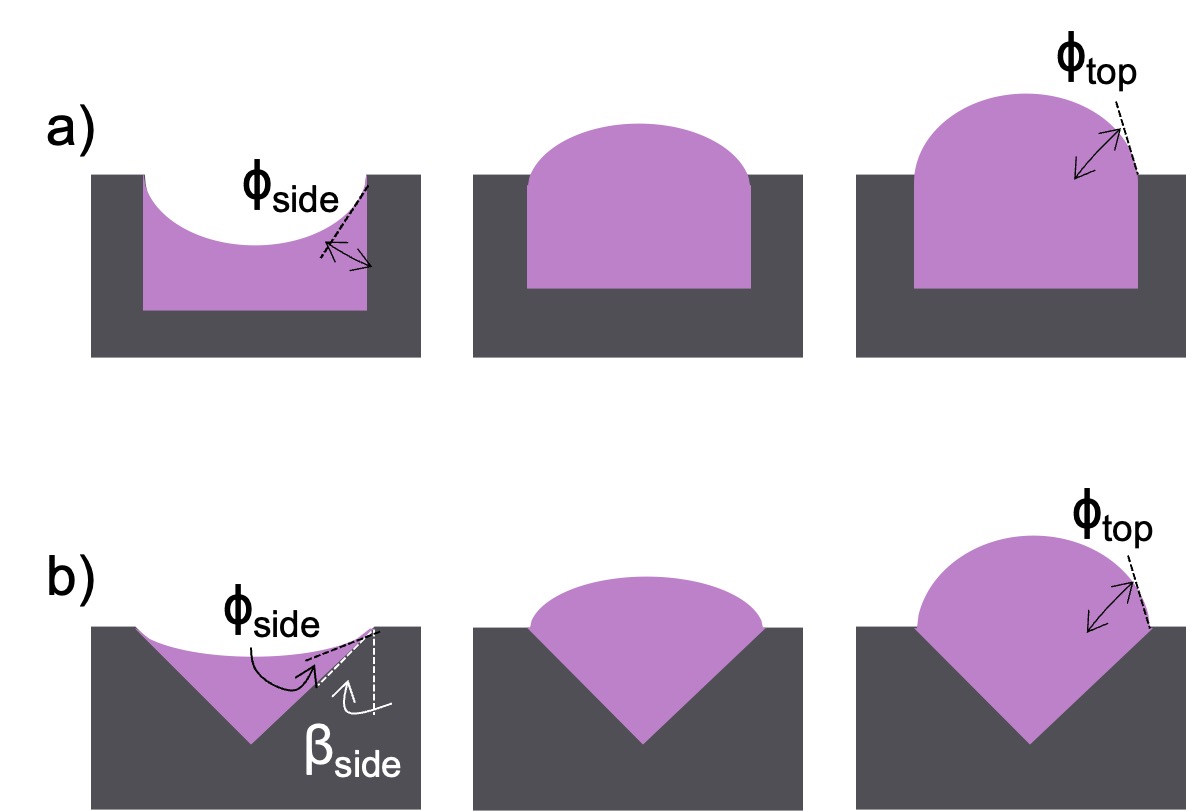

Supplement: Supplementary file 2 [file ot5c00373_si_002.zip › SI2 ot5c00373/SI/Images/FigureS5.jpg]

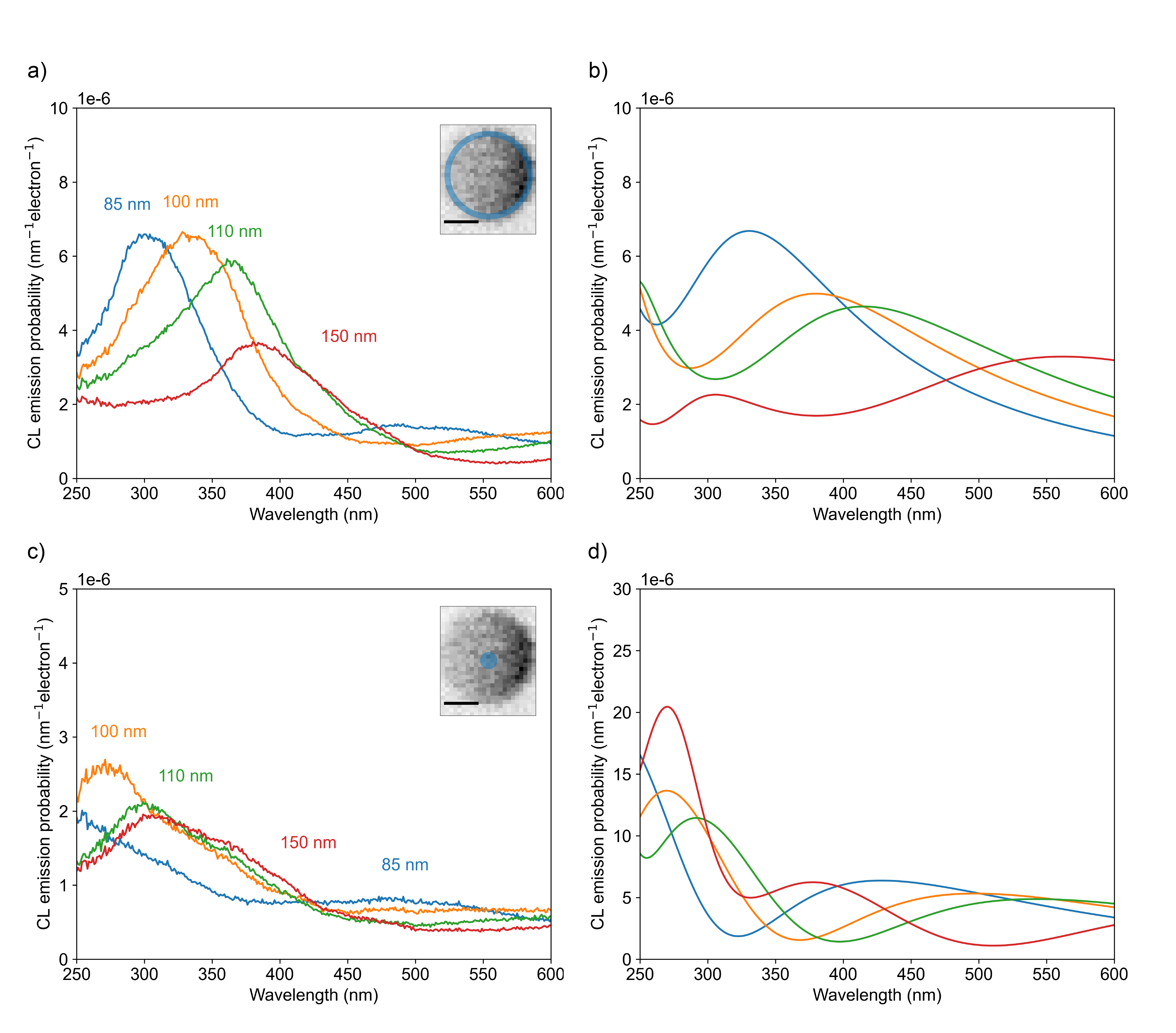

Supplement: Supplementary file 2 [file ot5c00373_si_002.zip › SI2 ot5c00373/SI/Images/FigureS6.jpg]

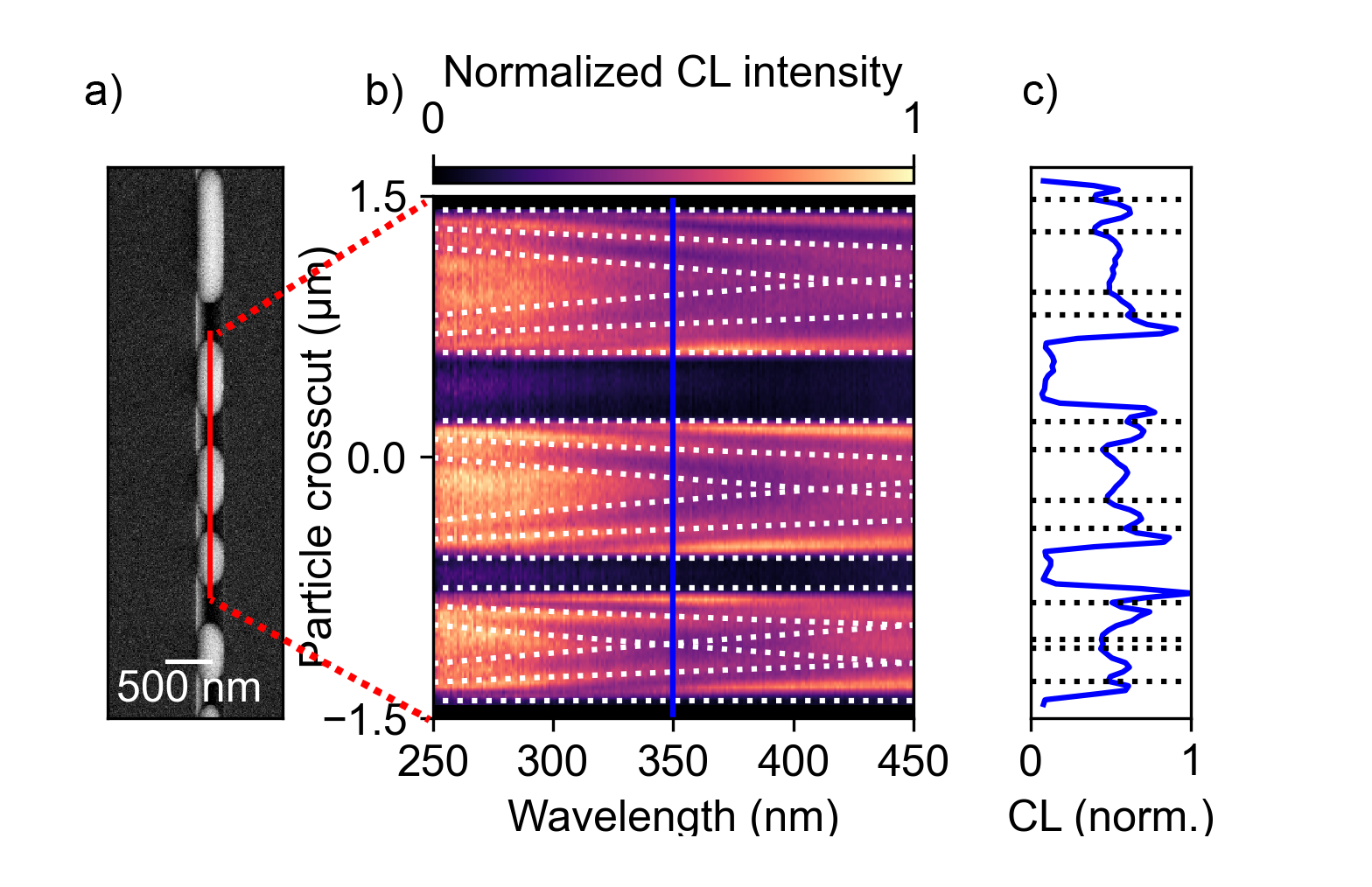

Supplement: Supplementary file 2 [file ot5c00373_si_002.zip › SI2 ot5c00373/SI/Images/FigureS7.png]

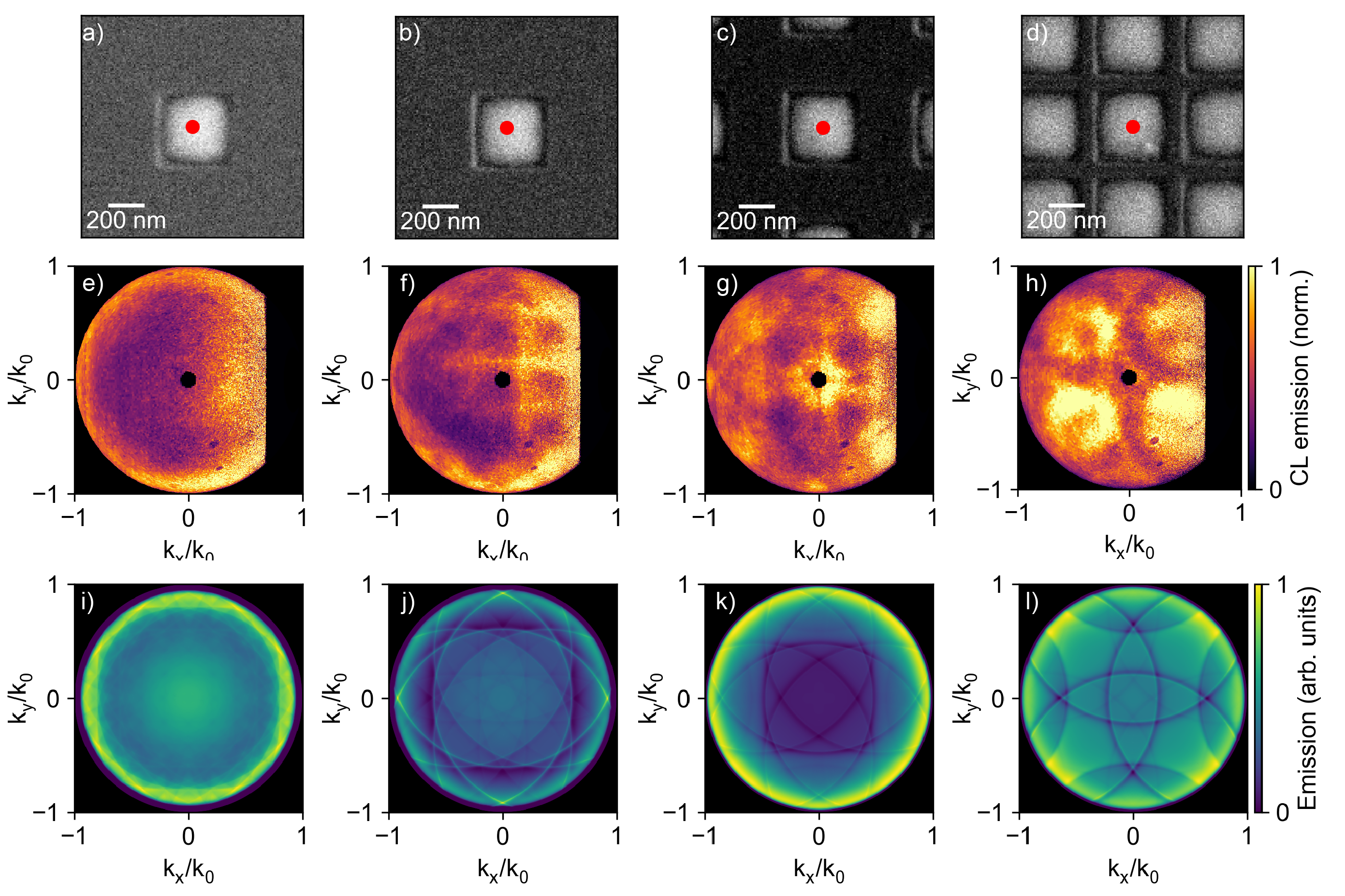

Supplement: Supplementary file 2 [file ot5c00373_si_002.zip › SI2 ot5c00373/SI/Images/FigureS8.jpg]
